# Radioprotective Effect of Grape Seed Proanthocyanidins In Vitro and In Vivo

**DOI:** 10.1155/2016/5706751

**Published:** 2016-06-26

**Authors:** Yijuan Huang, Hainan Zhao, Kun Cao, Ding Sun, Yanyong Yang, Cong Liu, Jianguo Cui, Ying Cheng, Bailong Li, Jianming Cai, Fu Gao

**Affiliations:** ^1^Department of Radiology, The First Hospital of Jiaxing, 1882 Zhonghuan South Road, Jiaxing, Zhejiang 314000, China; ^2^Department of Radiation Medicine, Faculty of Naval Medicine, Second Military Medical University, 800 Xiangyin Road, Shanghai 200433, China

## Abstract

We have demonstrated that grape seed proanthocyanidins (GSPs) could effectively scavenge hydroxyl radical (•OH) in a dose-dependent manner. Since most of the ionizing radiation- (IR-) induced injuries were caused by •OH, this study was to investigate whether GSPs would mitigate IR-induced injuries in vitro and in vivo. We demonstrated that GSPs could significantly reduce IR-induced DNA strand breaks (DSBs) and apoptosis of human lymphocyte AHH-1 cells. This study also showed that GSPs could protect white blood cells (WBC) from IR-induced injuries, speed up the weight of mice back, and decrease plasma malondialdehyde (MDA), thus improving the survival rates of mice after ionizing radiation. It is suggested that GSPs have a potential as an effective and safe radioprotective agent.

## 1. Introduction

Ionizing radiation (IR) produces large amount of reactive oxygen species (ROS) such as hydroxyl radical (•OH), superoxide (O_2_^−^), and hydrogen peroxide (H_2_O_2_), among which •OH contributes 60–70% to IR-induced injuries [1]. IR exerts immediate biological effects by rapid reaction of ROS with cellular macromolecules like DNA, lipids, and proteins.

The sulphydryl compound amifostine, which is the only radioprotectant approved to be used in humans by FDA, has shown effective radioprotective ability [2]. However, the obvious side effects limit its clinical use, such as hypertension, nausea, and vomiting [3]. Considering the urgent requirements, we need to bring progress in the development of safe and effective therapeutic agents.

Previously, we have demonstrated that grape seed proanthocyanidins (GSPs) could effectively scavenge hydroxyl radical (•OH) in a dose-dependent manner [4]. In the current study, we investigated whether GSPs exerted radioprotective effect in vitro and in vivo.

## 2. Materials and Methods

### 2.1. Cell Culture and GSPs Treatment

Human lymphocyte AHH-1 cells (American Type Culture Collection, Manassas, VA) were cultured in RPMI 1640 (Invitrogen, CA) with 10% foetal bovine serum (FBS) and 1% penicillin-streptomycin-glutamine at 37°C in a 5% CO_2_ humidified chamber. Cells were treated with different concentrations of GSPs-rich PBS and then radiated with different doses of *γ*-ray immediately, according to the need of the present study.

### 2.2. Radiation

The resource of ^60^Co radiation facility was provided by the Radiation Center in Faculty of Naval Medicine, Second Military Medical University, China. AHH-1 cells were radiated with 8 Gy at a dose rate of 1 Gy/min. Mice were exposed to different doses of radiation, according to the need of the present study.

### 2.3. Cell Viability

AHH-1 cells were cultured in 96-well plates for 24 hrs and pretreated with or without GSPs-rich PBS at 5 mins before radiation and further cultured for 24 hrs after radiation. Cell viability was determined by Cell Counting Kit (Dojindo Laboratories, Kumamoto, Japan).

### 2.4. Apoptosis Assays for Cultured Cells

The level of the apoptosis was determined by Apoptosis Detection Kit (Bipec Biopharma, Massachusetts, MA). Treated cells were analysed by flow cytometry. Alternatively, apoptosis was determined by Hoechst 33258, fluorescein diacetate (FDA), and PI staining as described previously [5].

### 2.5. Comet Assay

The DNA strand breaks (DSBs) were measured by using single-cell gel electrophoresis (comet assay) based on the method of Dubner et al. [6].

### 2.6. Animals and GSPs Treatment

All the protocols of animal experiment were consistent with the Guide for Care and Use of Laboratory Animals published by the US NIH. Six-week-old male BALB/c mice (Chinese Academy of Sciences, China) were used in the experiments. The animal house was kept with unwavering temperature (24°C), and the mice were fed ad libitum. About 1 h before radiation, grape seed proanthocyanidins were administered to mice by gavage, which were provided by Pure-one Bio Technology Co. Ltd. (Shanghai, China). GSPs were maintained through drinking for 1 week after radiation.

### 2.7. Survival Assays

Survival rates of the mice were recorded daily for 30 days after radiation with 8 Gy at a dose rate of 1 Gy/min.

### 2.8. Body Weight

All mice were weighed by an electronic scale (GH-202, AND, Japan) at the same time of the day.

### 2.9. White Blood Cell

Caudal vein WBC was counted in cell count plate at the same time of the day as described previously [7].

### 2.10. MDA Assay

Arterial blood samples (0.6 mL) of the mice, which were collected 12 hrs after radiation, were taken for MDA assay with the method defined by Ohkawa et al. [8].

### 2.11. Statistical Analysis

Statistics were expressed as means ± SEM for each experiment and analysed by using One-Way Analysis of Variance. Between groups, a two-tailed Student's *t*-test was used. A *P* value less than 0.05 was considered to be statistically significant.

## 3. Results

### 3.1. GSPs-Rich PBS Increases Cell Viability of Radiated AHH-1 Cells

**Figure 1:**
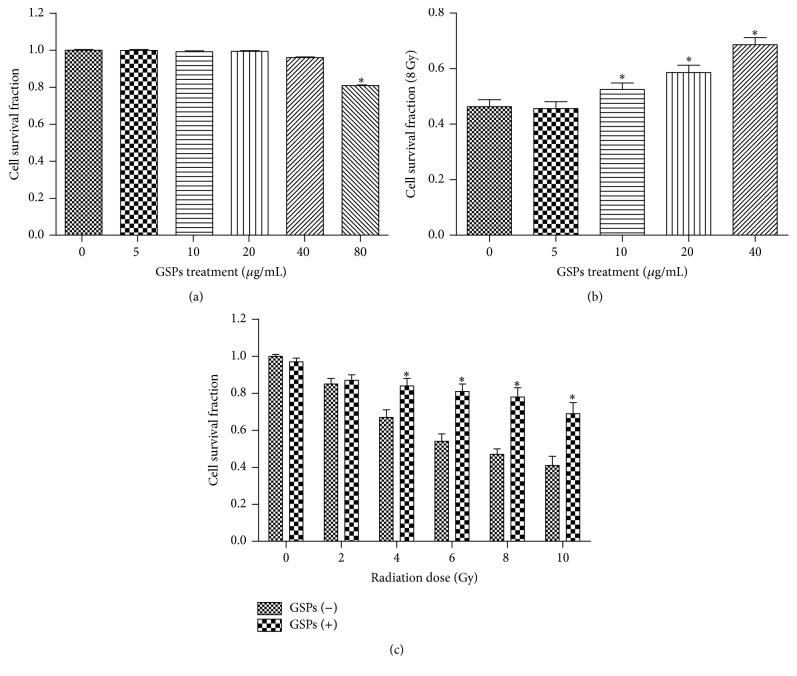
Grape seed proanthocyanidins (GSPs) mitigated radiation injuries on AHH-1 cells. (a) The cytotoxicity of different concentrations of GSPs against AHH-1 cells. (b) Dose-dependent effect of GSPs on cell viability induced by 8 Gy gamma radiation. (c) Variation of cell survival fractions pretreated with 40 *μ*g/mL GSPs before different doses of radiation. ^*∗*^ *P* < 0.05; *n* = 6.

Figure 1(a) showed that GSPs inhibited cellular proliferation and diminished the viability of AHH-1 cells when the concentration of GSPs was more than 80 *μ*g/mL. In addition, we demonstrated that pretreatment of AHH-1 cells with 5–40 *μ*g/mL GSPs-rich PBS before radiation significantly increased cell survival rates as compared to cells treated with radiation alone at all examined doses (up to 10 Gy), and the radioprotective effect of GSPs was dose-dependent (Figures 1(b) and 1(c)).

### 3.2. GSPs-Rich PBS Attenuated Apoptosis in Radiated AHH-1 Cells

**Figure 2:**
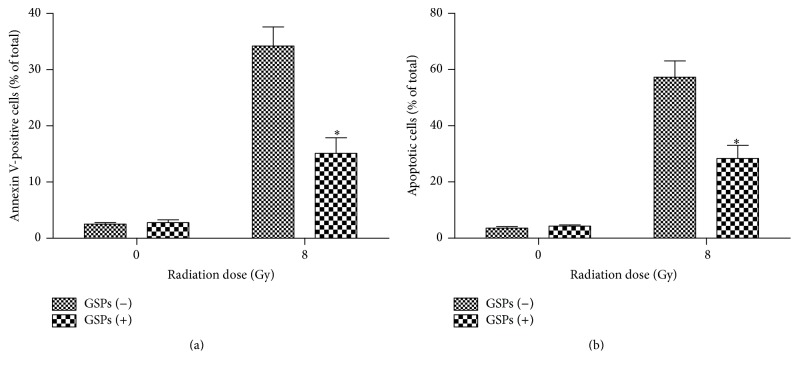
GSPs-rich PBS attenuated radiation-induced apoptosis in AHH-1 cells. (a) A bar graph of Annexin V-positive cells expressed as a percentage of total cells. Treated cells were collected 24 hrs after radiation, stained with Annexin V-APC and propidium iodide, and analysed by flow cytometry. (b) A bar graph of apoptotic cells expressed as a percentage of total cells. Cells were stained with FDA, Hoechst 33258, and PI 24 hrs after radiation and apoptotic cells were counted in multiple randomly selected fields. ^*∗*^ *P* < 0.05; *n* = 6.

The early apoptotic cells decreased when AHH-1 cells were pretreated with 40 *μ*g/mL GSPs-rich PBS as compared to cells pretreated with PBS alone in flow cytometry assay (Figure 2(a), 15.1% versus 34.2%, resp.). These findings were corroborated by the morphology of dying cells using Hoechst 33258, fluorescein diacetate, and propidium iodide staining. Radiated AHH-1 cells pretreated with GSPs-rich PBS had a reduced apoptosis of 28.4% as compared to 57.3% in PBS pretreated radiated cells (Figure 2(b)). These data suggest that GSPs attenuate apoptosis in radiated AHH-1 cells.

### 3.3. Comet Assay

**Figure 3:**
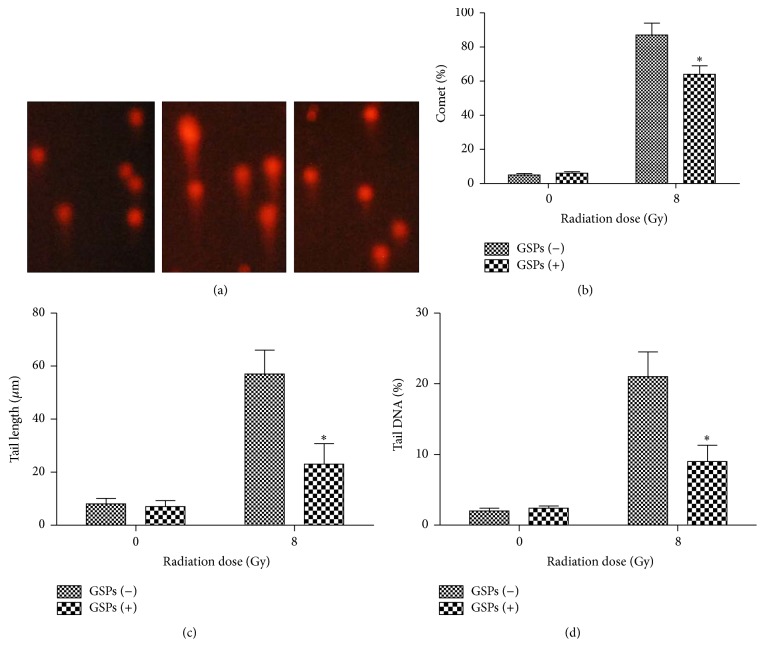
Effect of GSPs in AHH-1 cells on IR-induced DNA strand breaks assayed by comet assay. (a) Representative micrographs. (b) Comet%. (c) Tail length. (d) Tail DNA%. ^*∗*^ *P* < 0.05; *n* = 6.

The radiated mice resulted in an increase in the levels of all comet parameters (comet%, tail length, and tail DNA%), whereas pretreatment of GSPs before radiation inhibited the increase of these parameters significantly, indicating the protective effect of GSPs on IR-induced DNA damage (Figure 3).

### 3.4. Survival Rates of Mice

**Figure 4:**
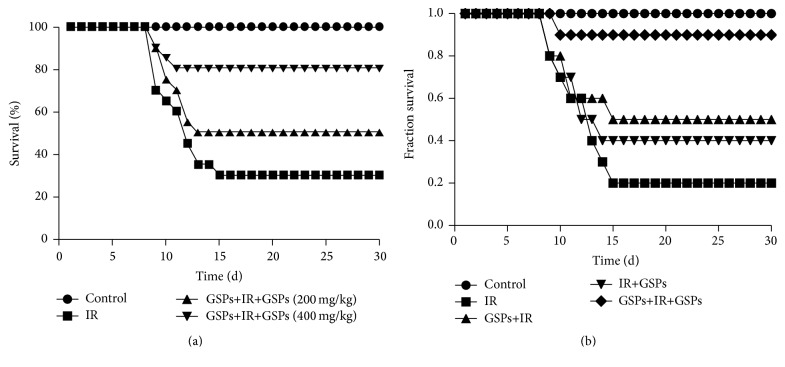
Survival rates of mice. (a) Different dosages of GSPs were given by gavage about 1 h before radiation and maintained through drinking until 1 week after radiation. (b) Different effects of the three strategies of GSPs (400 mg/kg of the body weight) delivery including therapy (after radiation, survival 40%), prevention (before radiation, survival 50%), and the combination of therapy and prevention medication (after radiation and before radiation, survival 80%). Representative results from one of three independent experiments are shown. ^*∗*^ *P* < 0.05; control, *n* = 10; others, *n* = 20.

70% of radiated mice without GSPs treatment died by the 15th day after radiation, while 50% of the mice survived when treated with 200 mg/kg of the body weight GSPs and 80% survived when treated with 400 mg/kg of the body weight GSPs (Figure 4(a)).  Figure 4(b) showed the different effects of the three strategies of GSPs (400 mg/kg of the body weight) delivery including therapy (after radiation, survival 40%), prevention (before radiation, survival 50%), and the combination of therapy and prevention medication (after and before radiation, survival 80%). We found that the combined medication improved the survival rate of the mice most.

### 3.5. Effect of GSPs on Body Weight of Mice after Radiation (5 Gy)

**Figure 5:**
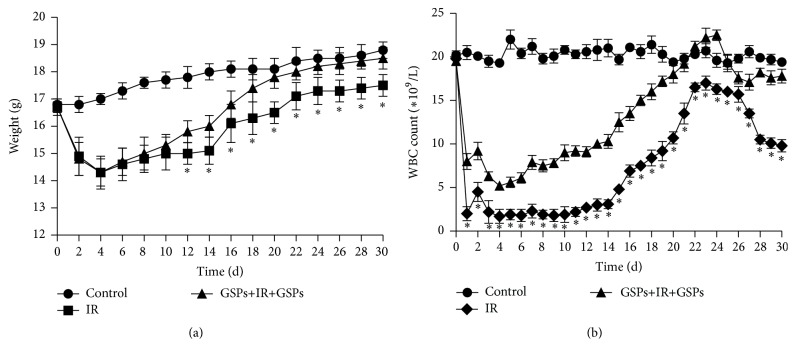
Effects of GSPs on (a) body weight and (b) WBC count of mice after radiation (5 Gy). Representative results from one of three independent experiments are shown. ^*∗*^ *P* < 0.05; for each group, *n* = 10.

Weight of mice fell sharply within 4 days after radiation, and GSPs cannot slow down its decrease (Figure 5(a)). However, in the subsequent time period, weight of mice began to increase, and GSPs significantly speeded up the weight back.

### 3.6. Effect of GSPs on WBC Count after Radiation (5 Gy)

GSPs slowed down the decrease of WBC count after radiation and speeded up the recovery of WBC count from the 4th day after radiation, while WBC count remained at lower levels until the 11th day after radiation in the radiation alone group (Figure 5(b)). WBC count was back up to the peak at the 24th day after radiation and then fell to a stable level. However, WBC count remained at higher levels in the GSPs-treated group than in the radiation alone group at the end of the experiment.

### 3.7. Changes in the Levels of Plasma MDA

**Figure 6:**
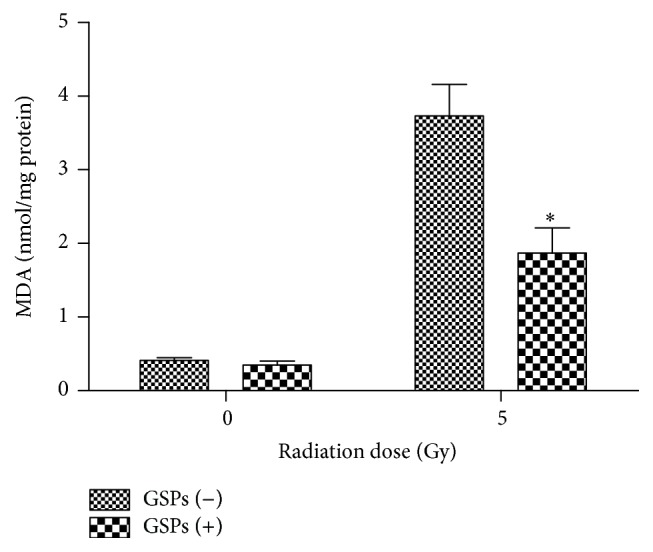
GSPs-rich saline significantly decreased levels of plasma MDA, a marker of oxidative stress. Representative results from one of three independent experiments are shown. ^*∗*^ *P* < 0.05; for each group, *n* = 3.

The plasma MDA concentrations were measured at 12 hrs after 5 Gy radiation (Figure 6). Plasma MDA concentrations in the GSPs-treated group were significantly lower than that in the radiation alone group. Changes in the levels of MDA indicate the antioxidant potential of GSPs.

## 4. Discussion

An immediate effect of radiation is the generation of reactive oxygen species (ROS) that can result in oxidative deterioration of DNA, lipids, and proteins [9]. ROS are also generated by inflammatory cells through induction of a cascade of cytokine activity [10] and from damaged mitochondria via leakage from the electron transport chain [11]. The fact that antioxidants were effective in ameliorating ionizing radiation-induced oxidative injuries has been demonstrated [12, 13]. Previously, we have assessed that GSPs exhibited significant free radical scavenging ability in a cell-free Fenton system [4]. In the current study, we demonstrated that GSPs protected AHH-1 cells and mice from IR-induced injury, which may result from their radical scavenging effect.

Membrane lipids are the major targets of ROS and the free radical chain reaction [14]. The increase of lipid peroxidation products, such as malondialdehyde, is the index of lipid damage [6]. Also, DNA is one of the major targets of ROS [15]. One of the consequences of DNA damage is apoptosis of the cells [9]. In our study, we observed a significant increase in the levels of plasma MDA and severe DNA strand breaks when exposed to ionizing radiation. However, pretreatment of GSPs prior to radiation exposure decreased the levels of MDA and mitigated DNA strand breaks, thus attenuating apoptosis of the cells.

Weight is one of the important indices to measure the quality of life. Ionizing radiation leads to disorders such as dyspepsia and thus results in weight loss [16]. In the current study, we observed that the GSPs-treated group had a significant increase in body weight compared to the radiation alone group, indicating that GSPs improved the digestion and absorption function of radiated mice, thereby increasing body weight of mice.

The lowest level of WBC after radiation plays the decisive role in the survival of radiated mice. IR-induced WBC decrease can result in life-threatening infections [17]. In this study, we observed that GSPs significantly increase the lowest level of WBC after radiation, indicating that GSPs could protect hematopoietic system from IR-induced damage, which may account for the significant increased survival rate in the GSPs-treated group.

In order to find a radioprotectant of real worth, we should consider the following features, such as bioavailability, safety and toxicity, distribution, and free radical scavenging ability. It is much to be regretted that WR-2721 is the only radioprotectant registered in use for humans. However, it has relatively high toxicity limiting its clinical use [18]. And cytokines and immunomodulators are effective to be used only when suffering from low radiation doses [19].

GSPs, widely available in grapes, have been reported to possess greater free radical scavenging ability and better protection against IR-induced lipid peroxidation and DNA damage than vitamins C and E, singly and in combination [20]. Previously, we have assessed the dose-dependent antioxidant ability of GSPs by ESR. In addition, it is important to note that GSPs can remain in the plasma and tissues for up to 7–10 days and exert antioxidant properties with no toxicity [21, 22]. Given these properties, GSPs have great potential as a clinical therapeutic agent.

In this study, we demonstrated that GSPs had a radioprotective effect in vitro and in vivo. The radical scavenging ability plays an important role in the radioprotective effect of GSPs. However, the exact mechanism remains to be studied.
